# Data Management in Multicountry Consortium Studies: The Enterics For Global Health (EFGH) *Shigella* Surveillance Study Example

**DOI:** 10.1093/ofid/ofad573

**Published:** 2024-03-25

**Authors:** Erika Feutz, Prasanta K Biswas, Latif Ndeketa, Billy Ogwel, Uma Onwuchekwa, Golam Sarwar, Shazia Sultana, Pablo Peñataro Yori, Alyssa Acebedo, Naveed Ahmed, Imran Ahmed, Hannah E Atlas, Alex O Awuor, Md Amirul Islam Bhuiyan, Bakary Conteh, Oualy Diawara, Sarah Elwood, Moussa Fane, Md Ismail Hossen, Mahzabeen Ireen, Abdoulie F Jallow, Mehrab Karim, Margaret N Kosek, Karen L Kotloff, Clement Lefu, Jie Liu, Rebecca Maguire, Farah Naz Qamar, Maureen Ndalama, John Benjamin Ochieng, Caleb Okonji, Loyda Fiorella Zegarra Paredes, Patricia B Pavlinac, Karin Perez, Sonia Qureshi, Francesca Schiaffino, Moussa Traore, Kirkby D Tickell, Richard Wachepa, Desiree Witte, Jennifer Cornick, M Jahangir Hossain, Farhana Khanam, Maribel Paredes Olortegui, Richard Omore, Samba O Sow, Mohammad Tahir Yousafzai, Sean R Galagan

**Affiliations:** Department of Global Health, University of Washington, Seattle, Washington, USA; International Centre for Diarrhoeal Disease Research, Bangladesh, Dhaka, Bangladesh; Malawi Liverpool Wellcome Programme, Blantyre, Malawi; Liverpool School of Tropical Medicine, Liverpool, UK; Institute of Infection, Veterinary and Ecological Sciences, University of Liverpool, Liverpool, UK; Kenya Medical Research Institute, Center for Global Health Research (KEMRI-CGHR), Kisumu, Kenya; Centre pour le Développement des Vaccins du Mali (CVD-Mali), Bamako, Mali; Medical Research Council Unit The Gambia at the London School of Hygiene and Tropical Medicine, Fajara, The Gambia; Department of Pediatrics and Child Health, The Aga Khan University, Karachi, Pakistan; Division of Infectious Diseases and International Health, University of Virginia, Charlottesville, Virginia, USA; American Association for Cancer Research, Philadelphia, Pennsylvania, USA; Department of Pediatrics and Child Health, The Aga Khan University, Karachi, Pakistan; Department of Pediatrics and Child Health, The Aga Khan University, Karachi, Pakistan; Department of Global Health, University of Washington, Seattle, Washington, USA; Kenya Medical Research Institute, Center for Global Health Research (KEMRI-CGHR), Kisumu, Kenya; International Centre for Diarrhoeal Disease Research, Bangladesh, Dhaka, Bangladesh; Medical Research Council Unit The Gambia at the London School of Hygiene and Tropical Medicine, Fajara, The Gambia; Centre pour le Développement des Vaccins du Mali (CVD-Mali), Bamako, Mali; Division of Infectious Diseases and International Health, University of Virginia, Charlottesville, Virginia, USA; Centre pour le Développement des Vaccins du Mali (CVD-Mali), Bamako, Mali; International Centre for Diarrhoeal Disease Research, Bangladesh, Dhaka, Bangladesh; International Centre for Diarrhoeal Disease Research, Bangladesh, Dhaka, Bangladesh; Medical Research Council Unit The Gambia at the London School of Hygiene and Tropical Medicine, Fajara, The Gambia; Medical Research Council Unit The Gambia at the London School of Hygiene and Tropical Medicine, Fajara, The Gambia; Division of Infectious Diseases and International Health, University of Virginia, Charlottesville, Virginia, USA; Center for Vaccine Development and Global Health, University of Maryland School of Medicine, Baltimore, Maryland, USA; Department of Pediatrics, University of Maryland School of Medicine, Baltimore, Maryland, USA; Department of Medicine, University of Maryland School of Medicine, Baltimore, Maryland, USA; Malawi Liverpool Wellcome Programme, Blantyre, Malawi; School of Public Health, Qingdao University, Qingdao, China; Center for Vaccine Development and Global Health, University of Maryland School of Medicine, Baltimore, Maryland, USA; Department of Pediatrics and Child Health, The Aga Khan University, Karachi, Pakistan; Malawi Liverpool Wellcome Programme, Blantyre, Malawi; Kenya Medical Research Institute, Center for Global Health Research (KEMRI-CGHR), Kisumu, Kenya; Kenya Medical Research Institute, Center for Global Health Research (KEMRI-CGHR), Kisumu, Kenya; Asociación Benéfica Prisma, Iquitos, Loreto, Peru; Department of Global Health, University of Washington, Seattle, Washington, USA; Asociación Benéfica Prisma, Iquitos, Loreto, Peru; Department of Pediatrics and Child Health, The Aga Khan University, Karachi, Pakistan; Division of Infectious Diseases and International Health, University of Virginia, Charlottesville, Virginia, USA; Faculty of Veterinary Medicine, Universidad Peruana Cayetano Heredia, Lima, Peru; Centre pour le Développement des Vaccins du Mali (CVD-Mali), Bamako, Mali; Department of Global Health, University of Washington, Seattle, Washington, USA; Malawi Liverpool Wellcome Programme, Blantyre, Malawi; Malawi Liverpool Wellcome Programme, Blantyre, Malawi; Institute of Infection, Veterinary and Ecological Sciences, University of Liverpool, Liverpool, UK; Institute of Infection, Veterinary and Ecological Sciences, University of Liverpool, Liverpool, UK; Medical Research Council Unit The Gambia at the London School of Hygiene and Tropical Medicine, Fajara, The Gambia; International Centre for Diarrhoeal Disease Research, Bangladesh, Dhaka, Bangladesh; Asociación Benéfica Prisma, Iquitos, Loreto, Peru; Kenya Medical Research Institute, Center for Global Health Research (KEMRI-CGHR), Kisumu, Kenya; Centre pour le Développement des Vaccins du Mali (CVD-Mali), Bamako, Mali; Department of Pediatrics and Child Health, The Aga Khan University, Karachi, Pakistan; Department of Global Health, University of Washington, Seattle, Washington, USA

**Keywords:** clinical research, consortium studies, data management, data quality, population enumeration

## Abstract

**Background:**

Rigorous data management systems and planning are essential to successful research projects, especially for large, multicountry consortium studies involving partnerships across multiple institutions. Here we describe the development and implementation of data management systems and procedures for the Enterics For Global Health (EFGH) *Shigella* surveillance study—a 7-country diarrhea surveillance study that will conduct facility-based surveillance concurrent with population-based enumeration and a health care utilization survey to estimate the incidence of *Shigella­*-associated diarrhea in children 6 to 35 months old.

**Methods:**

The goals of EFGH data management are to utilize the knowledge and experience of consortium members to collect high-quality data and ensure equity in access and decision-making. During the planning phase before study initiation, a working group of representatives from each EFGH country site, the coordination team, and other partners met regularly to develop the data management systems for the study.

**Results:**

This resulted in the Data Management Plan, which included selecting REDCap and SurveyCTO as the primary database systems. Consequently, we laid out procedures for data processing and storage, study monitoring and reporting, data quality control and assurance activities, and data access. The data management system and associated real-time visualizations allow for rapid data cleaning activities and progress monitoring and will enable quicker time to analysis.

**Conclusions:**

Experiences from this study will contribute toward enriching the sparse landscape of data management methods publications and serve as a case study for future studies seeking to collect and manage data consistently and rigorously while maintaining equitable access to and control of data.

## Teaser

This paper describes the data management systems and processes employed by the Enterics for Global Health: *Shigella* Surveillance Study (EFGH). Systems and procedures were designed to promote equity in access and decision-making while maintaining high-quality data standards.

While publishing scientific methods and results in peer-reviewed journals is expected and often obligatory, publications summarizing data management methods and experiences are less common. Publishing study findings can provide standardization or guidance for future research methodology. Lack of publication on data management strategies can lead future research teams to continually reinvent methods rather than learn from past experiences. This risks poor data quality, especially for complex studies where robust data management is crucial. This dearth of shared knowledge may ultimately hinder science's ability to advance health interventions for preventing and treating diseases.

It is becoming increasingly common for funding agencies to require open-access availability of analysis data sets and submission of written data management plans alongside grant applications. Many reports summarize guiding principles for data, but fewer describe the specific processes utilized in individual clinical research studies. Kanza and Knight (2022) provide an excellent description of 10 data management guidelines [1], 1 of which emphasizes the need for data sets to be findable, accessible, interoperable, and reusable (FAIR) to reduce barriers to data reuse and team science, a continued necessity alongside increasing requirements for data sharing [1, 2]. However, to adhere to FAIR principles, strong data management is crucial and cannot be learned from guidelines alone. Biswas et al. (2012) reported the data systems, flow, and major challenges of the Global Enteric Multicenter Study (GEMS), a large, multicountry enteric health project that set a precedent for publishing data management procedures of multicenter enteric projects [3, 4]. Ultimately, the best way to develop robust data management practices is to learn from the specifics of challenges and successes in similar studies.

The Enterics For Global Health *Shigella* surveillance study (EFGH) is a 24-month diarrhea surveillance study aimed at determining the incidence of *Shigella*-attributed diarrhea in children 6 to 35 months of age residing in 7 countries in populations whose demographic and health care utilization characteristics are well described [5–13]. This large consortium study has 2 components: diarrhea case surveillance (DCS) and population enumeration with the health care utilization survey (PEHUS). The DCS component will enroll children presenting with diarrhea at select medical facilities in Dhaka, Bangladesh; Siaya County, Kenya; Blantyre, Malawi; Bamako, Mali; Karachi, Pakistan; Iquitos, Peru; and Basse, The Gambia. Microbiological and molecular testing will be used to detect *Shigella* spp. in stool at enrollment. Clinical, sociodemographic, and anthropometric data will be collected at enrollment, week 4, and month 3 follow-up visits [6]. Concurrently, the PEHUS component will use randomized cluster sampling to enumerate the population living in the catchment areas of the health facilities. Children's diarrhea history and health care-seeking behavior will be surveyed to establish the population at risk and calculate a health care–seeking adjustment [5].

This complex, multicountry study requires robust data management practices. The goals of data management in EFGH are to ensure readily available and high-quality data, to ensure equity across the consortium in access and decision-making, and to utilize the vast knowledge and experience of EFGH consortium members. These goals guided the development of EFGH data management systems, quality control processes, reporting and monitoring strategies, data access and security, and finalization. Here we describe the development and implementation of EFGH data management systems and processes.

## DESIGN, TRAINING, AND PREPARATION

The EFGH data team consists of members from the coordination team at the University of Washington and from each of the 7 EFGH country site teams. Site data team members manage day-to-day data collection activities. Team members of some sites will be directly involved in data collection at facilities or in the community, while others will monitor data collection as it arrives from the daily activities. Site data team members are responsible for establishing data collection and quality assurance (QA) procedures at their site to ensure accurate data collection and timely data entry. Coordination data team members manage cross-site operations and databases to maintain consistency in data collection methods and management across the consortium and to support site data management leads.

Before developing data systems, a Data Management Working Group (DMWG) was formed with coordination and site data team members to make decisions about standardized data management activities in EFGH. Using the team's broad experiences from other large multicenter studies such as the Childhood Acute Illness and Nutrition (CHAIN) network [14], the Antibiotics for Children with Diarrhea (ABCD) study [15], GEMS [4, 16], and others, the DMWG developed a data management plan (DMP) to establish standard operating procedures surrounding data collection and management and to set data quality standards. The DMP also includes a schedule for the coordination data team-produced quality checks, data exports and reporting, and specific data security expectations. In anticipation of changing data management needs upon study initiation, the coordination data team will begin virtual data management office hours to provide a space for team members to bring site-specific or cross-consortium issues to the coordination team for technical consultation and discussion. Office hours will be scheduled twice monthly and will provide a space for ongoing communication among all team members throughout the study.

Standardized data management training was developed and hosted online using Canvas (Instructure, Inc., Salt Lake City, UT, USA). These modules provide training materials and will remain an ongoing resource accessible to all team members. Modules were developed by both coordination and site data team members focusing on data collection and entry in the databases, study dashboards, reporting schedule and structure, and quality control and assurance procedures. Additionally, an identical database was created for site teams to practice data entry and become familiar with the database systems used in EFGH. The training database will remain available throughout the study for training new team members.

## DATA SYSTEMS AND FLOW

**Figure 1.**
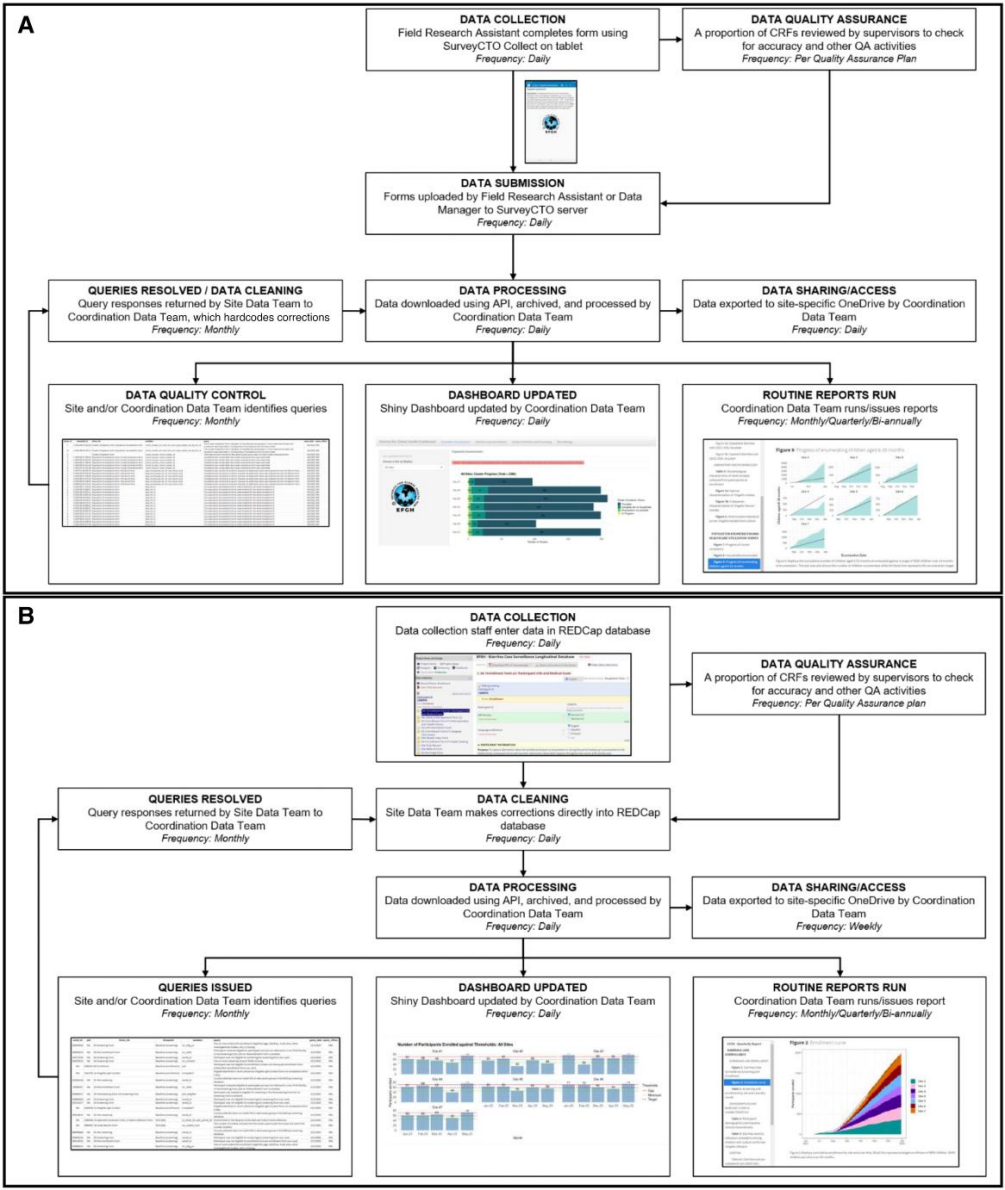
Data flow process for population enumeration and health care utilization survey data (A) and diarrhea case surveillance data (B). Abbreviations: CRF, case report forms; QA, quality assurance.

Data collection and processes vary between the DCS and PEHUS components of the study, as shown in Figure 1A and B. Collection and entry of DCS data will utilize the Research Electronic Data Capture (REDCap) system (Vanderbilt University, Nashville, TN, USA) hosted at the University of Washington [17, 18], and PEHUS data will be collected using SurveyCTO (Dobility, Inc., Cambridge, MA, USA), a mobile data collection system utilizing the Open Data Kit (ODK) platform. REDCap is a versatile, web-based data capture application supporting a mobile application for offline use [19]. The system supports useful features such as simple branching logic, data entry validation, automated alerts, and highly customizable user and data access rights. REDCap also maintains audit trails for tracking changes to data fields and has customizable modules for building study reports and developing automated data quality checks. Data from REDCap can be easily exported in varying formats for data analysis, and there is application programming interface (API) access for data to be directly pulled into statistical software. However, REDCap has some limitations as only simple skip logic and calculated fields are available, resulting in customization barriers for complex studies like EFGH [17, 18].

SurveyCTO is a mobile data collection platform designed for offline settings where the network signal may be unavailable or unstable. Data are collected using the SurveyCTO Collect application on a smartphone or tablet, stored locally on the device, and then submitted to the SurveyCTO server once a network is available. SurveyCTO offers encryption and redundancy for data collection security. The system supports similar features to REDCap such as branching logic, data entry validation, and API access. SurveyCTO also includes advanced functionality like randomized elements, more customizable calculated fields, and variable looping for complex survey designs (such as a household-level form that repeats individual-level questions depending on the number of household residents entered in a previous question). SurveyCTO can publish to and access data from server data sets, allowing for workflows where forms automatically update themselves as data are collected and forms are synced with the server. Drawbacks of SurveyCTO include the inability to limit user privileges to site-specific data without additional purchase, the risk of data loss should a device break or be misplaced before submission of data, and limited flexibility in export formats.

EFGH DCS data will be collected at each recruitment center by the EFGH clinical team, and PEHUS data will be collected in communities by field research assistants. Data for screening, enrollment, and follow-up in DCS will be collected in real time either by directly entering data into REDCap electronic Case Report Forms (eCRFs; Bangladesh, Kenya, Mali, and The Gambia sites) or using paper Case Report Forms (pCRFs), to then be entered daily into REDCap (Peru and Malawi sites). The Pakistan team will utilize offline eCRF data collection using the REDCap Mobile application. pCRFs and eCRFs contain the same fields, and all sites collect the same data, except for necessary minor differences in site-specific questions. Internet accessibility at facilities and team preference determined whether facilities will use eCRFs or pCRFs. As data errors are identified through QA activities, changes will be made by site data entry teams directly within the REDCap database. REDCap supports multilanguage functionality (teams can toggle forms between languages programmed into the database) [17], and the EFGH Mali and Peru teams enter data using French and Spanish interfaces, respectively. However, translations are labor-intensive as they must be manually input for each question, answer choice, and for all interface items, and translations are not compatible with REDCap Mobile.

The PEHUS eCRFs will be loaded and all data collected in real time using the SurveyCTO Collect application on tablets at each site. At the end of each day, completed eCRFs will be synced from each device to the SurveyCTO server. Some teams will utilize a workflow in which a supervisor reviews forms to check for errors before finalizing and submitting to the server. Like REDCap, SurveyCTO provides multilanguage functionality that allows teams to toggle between languages, and the Mali, Pakistan, and Peru teams can collect data using eCRFs in French, Urdu, and Spanish, respectively. While less labor-intensive than inputting translations in REDCap, translations still must be manually entered into the XLSForm definition and require plugins to support languages such as Urdu that are read from right to left.

While most laboratory data will be stored in the REDCap database, TaqMan Array Card (TAC) data will be stored separately. Each site laboratory team will conduct testing using the TAC enteric pathogen panel [20] and periodically upload the data into a MuSIC (Multi-Schema Information Capture) database (University of Virginia [UVA], Charlottesville, VA, USA). The MuSIC server is housed securely within the Clinical Data Repository in the UVA data center. It provides industry best practices in system administration, backup, and disaster recovery. MuSIC supports secure data entry via encrypted forms, batch upload of spreadsheets and delimited text files, and download of data as spreadsheets, delimited text files, and SQLite databases. The Molecular Laboratory Coordination support unit at UVA manages the TAC data and conducts additional QA activities before providing data to the coordination data team on a quarterly basis [21].

The coordination data team will process raw data into operational data sets from daily REDCap and SurveyCTO exports and use these data sets to generate data queries, reports, and dashboards and create site-specific data sets to be shared daily with site data teams (for PEHUS) or weekly (for DCS) using the Microsoft OneDrive application (Microsoft Inc., Redmond, WA, USA). All data processing, reporting, and quality procedures will be conducted in R, version 4.3 (R Foundation for Statistical Computing, Vienna, Austria). Additionally, many site data teams will have their own systems for downloading and processing data and running internal reports. Panel 1 demonstrates the EFGH The Gambia team's data processing strategy.

**Panel 1.**
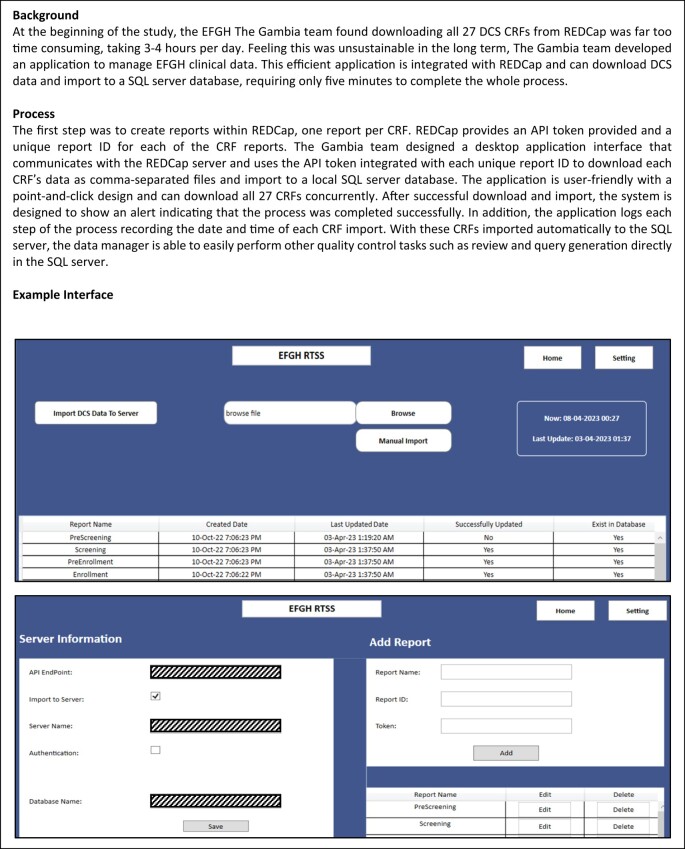
CASE STUDY: Data processing and computing at the EFGH The Gambia site. Abbreviations: CRF, Case report forms; DCS, Diarrhea case surveillance; EFGH, Enterics for Global Health.

## DATA QUALITY

Data quality refers to the correctness and completeness of data as well as the consistency and availability in a data system, and the extent to which the data are fit for their purpose [22]. Each EFGH site team developed an internal data QA plan to outline the routine and systematic processes that will be used to prevent and correct data errors to maximize and assess data quality and ensure that collected data accurately reflect the primary source. A summary of the site QA activities is shown in Table 1.

**Table 1.** Quality Assurance Activities Planned by EFGH Study Site and Activity

Study Site	Study Domain	Quality Assurance Activities^a^
Bangladesh	Population enumeration/health care utilization survey	Re-interviews (10%); review of fieldworker logbook; verification of clusters with 0 population; review of cluster completion eCRFs against logbooks.
	Diarrhea case surveillance	Review of paper laboratory forms against data entered in REDCap; sit-in interviews.
Kenya	Population enumeration/health care utilization survey	Sit-in interviews (5% of interviews per cluster); re-interviews (5% of interviews per cluster); review of completed forms before submission; review of cluster completion forms against logbooks; review of informed consent forms.
	Diarrhea case surveillance	Sit-in interviews (10%); re-interviews (5%); verification of entered forms in REDCap; review of lab forms (paper) entered in REDCap; review of informed consent forms; review of hospital registry and study registration log; review of enrolled children in screening and longitudinal databases.
Malawi	Population enumeration/health care utilization survey	Review of cluster completion forms against logbooks; review of informed consent forms; verification of coordinates within clusters; household revisits.
	Diarrhea case surveillance	Verification of entered forms in REDCap (30%–50%); review of paper forms (30%–50%).
Mali	Population enumeration/health care utilization survey	Review of cluster completion forms against logbooks (15%); review of informed consent forms; re-interviews (5%).
	Diarrhea case surveillance	Sit-in interviews; verification of paper forms; review of paper forms entered into REDCap.
Pakistan	Population enumeration/health care utilization survey	Review of fieldworker logbook; review of completed forms before submission; verification of completed surveys and clusters with 0 population; sit-in interviews; re-interviews (5%).
	Diarrhea case surveillance	Sit-in interviews (10%); re-interviews (10%); review of lab forms (paper) entered into REDCap; review of registration log; diarrhea diary entry confirmation (30%); review of enrolled children in screening and longitudinal databases.
Peru	Population enumeration/health care utilization survey	Review of informed consent forms; review of cluster completion forms against logbooks; review of fieldworker logbook.
	Diarrhea case surveillance	Review of completed paper forms; sit-in interviews; review of paper forms entered into REDCap.
The Gambia	Population enumeration/health care utilization survey	Review of fieldworker logbook; review of cluster completion forms against logbooks; review of informed consent forms; sit-in interviews.
	Diarrhea case surveillance	Verification of entered forms in REDCap; review of lab forms (paper) entered in REDCap; review of paper forms entered into REDCap (10%); review of registration\clinical log; sit-in interviews.

Abbreviation: EFGH, Enterics For Global Health.

^a^Frequency of quality assurance activity is indicated as a percentage of enrolled participants where the activity is performed. Where no percentage is listed, the activity is to be performed for every participant.

Data cleaning code is being developed by both the coordination and site data teams to identify discrepancies (inconsistency in data, missing values, range checks, skip patterns, and protocol deviations) in collected data and generate query lists. The coordination data team will produce monthly query lists to be resolved by site teams within the month. Additionally, site data teams will produce site-specific queries. Query resolution will involve direct correction to databases (most DCS queries) or require the coordination data team to hardcode resolutions into data sets (for the PEHUS data and DCS queries that cannot be resolved in REDCap directly). Sites that use pCRFs for DCS data collection plan to employ manual comparisons of pCRFs against database entries. Panel 2 details specific procedures employed by the Malawi team to maintain high data quality in both eCRFs and pCRFs.

**Panel 2.**
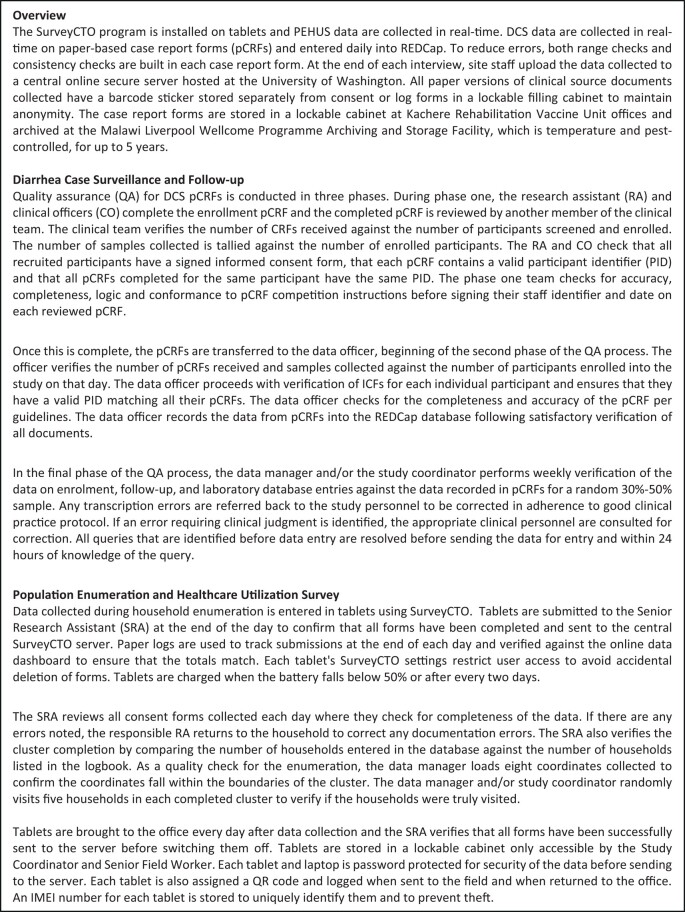
CASE STUDY: Data flow and quality assurance at the EFGH Malawi site. Abbreviations: CO, clinical officer; ICF, Informed Consent Form; pCRF, paper-based case report forms; PID, participant identifier; QA, quality assurance; RA, research assistant; SRA, senior research assistant.

In addition to the above QA procedures, the SurveyCTO and REDCap data collection systems have internal validations programmed within the data collection tools including the following: requiring most fields (to avoid missingness), validation checks (including only allowing certain characters or specific ranges for numeric fields), branching logic, banners alerting the data collector about a possible error, and calculated fields [6, 7]. As anthropometry measures are a significant component of enrollment data, these QA activities are standardized across sites and will include repeated measurements of enrolled participants, observation of measurements, routine calibration of weighing devices, standardization tests, and monitoring of baseline and follow-up measurements [6].

## REPORTING AND MONITORING

To ensure that all consortium members can track study progress and monitor data, routine data reports will be available to the site teams and collaborating partners on a daily, monthly, quarterly, and biannual basis. A data dashboard will be updated daily to allow near real-time monitoring of key study metrics such as the number of children screened and enrolled in DCS, the number of culture-positive *Shigella* cases, and the number of households and clusters enumerated during PEHUS. This data availability enables site teams to quickly identify and share findings with clinical staff and local policy-makers. The secure data dashboard uses the Shiny package in R (Posit, Inc., Boston, MA, USA) and will be hosted on the EFGH study website [23]. An example of the EFGH dashboard can be found at https://efgh.shinyapps.io/efgh_manuscript/.

All other reports will be produced as HTML files using R Markdown (Posit, Inc., Boston, MA, USA) [24, 25]. Monthly reports will be specific to each site and include summaries of key operational indicators such as reasons for screen-outs, study retention, and progress against enrollment and enumeration targets. Quarterly reports summarize data from across the consortium and allow for comparison across sites in terms of demographics, *Shigella* culture positivity, serotypes, antibiotic resistance, care-seeking behavior, and treatment-related costs. Unlike monthly reports, which will only be shared with the relevant site team, quarterly reports will be available to all site teams, study collaborators, and funding partners. Biannual reports will be produced to show preliminary estimates of the primary and secondary aims of the study to facilitate discussion among investigators, analysts, and funding partners about end points and analysis. All EFGH reports will be noneditable and password-protected as appropriate.

## DATA ACCESS, SHARING, AND SECURITY

EFGH prioritizes data security. All devices to be used for data collection are password-protected and encrypted, and both REDCap and SurveyCTO require a username and password to access. REDCap in particular supports user authentication and role-based privileges [18]. Tablets used for collecting PEHUS data will be stored in locked cabinets at each field site, and each site team has a locked data office to store pCRFs and consent forms and to house EFGH-dedicated computers. Collection and storage of identifiable data (such as initials for later identification of participants and dates of birth for inclusion criteria confirmation) and medical or operational records will be kept to a minimum. Participant records will be stored in locked cabinets with access limited to authorized study staff. The encrypted database data will be downloaded and archived to a Microsoft OneDrive server maintained by the University of Washington and accessible to all site data teams.

Collection of personally identifying information is avoided by using numeric participant and household identifiers. In DCS, screened children are assigned a 9-digit identifier (#-#-####-###) combining the country code (#), clinic code (#), a 4-digit sequential code (####), and the staff code (###). Those enrolled are assigned a pregenerated 7-digit participant ID (PID; #-#-####-#) using the code country (#), health facility code (#), a 4-digit sequential code (####), and a 1-digit Verhoeff check-digit (#), which is a matrix-based algorithm that reduces the number of single-digit errors and transpositions [26]. During PEHUS data collection, each household is assigned a 14-digit unique ID using the country code (#), date (YYYYMMDD), staff ID (###), and a 2-digit sequential ID starting at “01” each day (#-YYYYMMDD-###-##).

The coordination data team will have access to the data for all 7 countries, and each site will always have access to its own data. The coordination team will produce site-specific data sets to be shared with site teams via OneDrive daily and weekly for PEHUS and DCS, respectively. Site data teams have download rights to their site's DCS data from REDCap at their discretion; however, SurveyCTO does not support parsing data access by group without purchasing additional teams (which was determined to be cost-prohibitive for EFGH), so site teams will not have direct download privileges for PEHUS data. All consortium members can access the daily data dashboard and routine reports for internal monitoring.

## DATA FINALIZATION

Upon completion of data collection activities, data will need preparation for analysis. Extensive data cleaning will take place to ensure that analyses are performed with the highest quality data. Final data cleaning will mirror routine data quality checks but will allow no outstanding queries and will put specific attention on variables required for descriptive analyses and primary and secondary aims. Once cleaning is complete, databases will be locked to additional edits, and final data sets will be produced. Analytic data sets will be limited to the variables necessary for the analysis and shared with team members upon request. Upon publication of the primary manuscript, de-identified data sets will be shared with EFGH team members, and data used in the primary analysis will be published to Dataverse (Institute for Quantitative Social Science [IQSS], Harvard University, Cambridge, MA, USA) alongside publication of the primary manuscript.

## COMMENTARY

Data management in large consortium studies requires a flexible approach that utilizes each team's strengths and prior experiences while ensuring that the data are collected consistently and rigorously across the consortium. In the EFGH study, each data team is highly experienced, having participated in other large, high-profile consortium studies such as the Strategic Typhoid Alliance across Africa and Asia (STRATAA) [27], The Typhoid Vaccine Acceleration Consortium (TyVAC) [28], GEMS [16], the Malnutrition and Enteric Disease Study (MAL-ED) [29], the Vaccine Impact on Diarrhea in Africa Study (VIDA) [30], ABCD [15], and the Epidemiology Study of Malaria Transmission Intensity in Sub-Saharan Africa (EPI-MAL) [31], among others. While the coordination team was responsible for the final design and implementation of the data system, the DMWG provided collective input and feedback throughout the year-long planning period ahead of study initiation.

To align with EFGH's data management goals of ensuring equity and utilizing the wealth of experience across the consortium, adapting data management strategies to work within teams’ existing systems is crucial. For example, each site team will use their choice of data processing language to process data sets, and the coordination data team will output data sets in varying formats (.csv, .rds, .dta, etc.). Additionally, rather than mandating specific activities to ensure data quality and completeness, each EFGH team developed its own QA plan in consultation with the coordination data team. This activity provided an opportunity for cross-site collaboration and learning. Each team will use their favored strategy (pCRFs, direct eCRF data entry, or offline mobile app eCRF entry) for collecting and entering DCS data in REDCap. Finally, while the primary purpose of the DMWG was to develop the DMP and make decisions about data systems, this structure will remain in place throughout the study as a space for technical support and troubleshooting.

One important consideration in consortium studies is the trade-off between consistency and comparability between sites and opportunities for site-specific adaptations and ownership of data systems. While each data team has its preferred database systems and REDCap and SurveyCTO is new to some EFGH teams, standardizing the database is necessary to streamline the coordination data team's ability to track and process data as they are collected, manage version control as updates are made to CRFs, and reduce errors arising from having to transcribe eCRFs across multiple databases.

An additional consideration is maintaining site team data access throughout the study while ensuring that rigorous methods are employed to protect participant data. While equity in data access is important, broadly providing data access to all site data teams could enable data misconduct and potentially threaten data security. This consideration led to the decisions surrounding REDCap and SurveyCTO access for site teams. Because REDCap allows parsing data by site, teams have direct download access, whereas SurveyCTO data must be downloaded and processed daily by the coordination data team.

As described here, the challenges of data management in large, multicountry studies where each collaborating research team has a different set of strengths, resources, and challenges require significant planning to establish acceptable, feasible, and manageable systems for all teams without jeopardizing quality standards. Challenges will certainly arise throughout the course of the EFGH study despite the intention and forethought in designing data management systems. This paper, and subsequent EFGH publications, will contribute to the landscape of data management methods publications and aid in designing data management systems for future studies.
